# Cardiac Remodeling: Concepts, Clinical Impact, Pathophysiological Mechanisms and Pharmacologic Treatment

**DOI:** 10.5935/abc.20160005

**Published:** 2016-01

**Authors:** Paula S. Azevedo, Bertha F. Polegato, Marcos F. Minicucci, Sergio A. R. Paiva, Leonardo A. M. Zornoff

**Affiliations:** Faculdade de Medicina de Botucatu, São Paulo, SP - Brazil

**Keywords:** Ventricular Remodeling, Heart Failure, Medication Therapy Management, Ventricular Dysfunction / physiopathology

## Abstract

Cardiac remodeling is defined as a group of molecular, cellular and interstitial changes that manifest clinically as changes in size, mass, geometry and function of the heart after injury. The process results in poor prognosis because of its association with ventricular dysfunction and malignant arrhythmias. Here, we discuss the concepts and clinical implications of cardiac remodeling, and the pathophysiological role of different factors, including cell death, energy metabolism, oxidative stress, inflammation, collagen, contractile proteins, calcium transport, geometry and neurohormonal activation. Finally, the article describes the pharmacological treatment of cardiac remodeling, which can be divided into three different stages of strategies: consolidated, promising and potential strategies.

## Introduction

The term "remodeling" was used for the first time in 1982 by Hockman and Buckey, in a myocardial infarction (MI) model. This term was aimed to characterize the replacement of infarcted tissue with scar tissue.^1^ Janice Pfeffer was the first researcher to use the term remodeling in the current context, to describe the progressive increase of the left ventricular cavity in experimental model of MI in rats.^2^ The term was then used in some scientific articles on morphological changes following acute MI. In 1990, Pfeffer and Braunwald published a review on cardiac remodeling following MI, and the term was adopted to characterize morphological changes after infarction, particularly increase in the left ventricle.^3^ However, in the following years, the term "remodeling" has also been used to describe different clinical situations and pathophysiological changes. For this reason, in 2000, a consensus from an international forum on cardiac remodeling was published, which defined cardiac remodeling as a group of molecular, cellular and interstitial changes that clinically manifest as changes in size, shape and function of the heart resulting from cardiac injury.^4^ Although two types of cardiac remodeling were recognized during the forum - physiological (adaptive) remodeling and pathological remodeling - this article focuses on deleterious, pathological cardiac remodeling.

## Clinical Characterization

**Figure 1:**
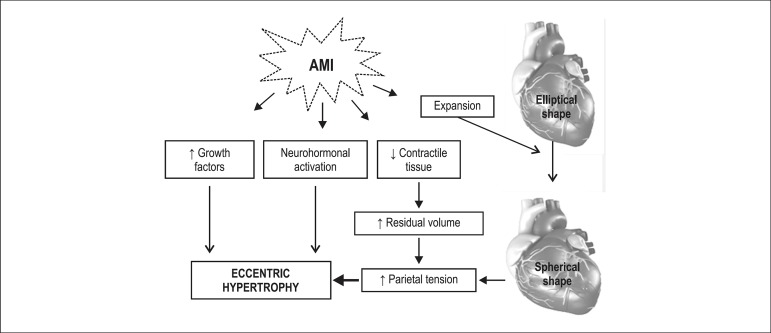
Left ventricular remodeling in the chronic phase of acute myocardial infarction (AMI).

The clinical diagnosis of remodeling is based on the detection of morphological changes - changes in the cavity diameter, mass (hypertrophy and atrophy), geometry (heart wall thickness and shape), areas of scar after MI, fibrosis and inflammatory infiltrate (e.g in myocardititis).^4^ The most used methods to detect these changes are echocardiography, ventriculography, and nuclear magnetic resonance^5^. One example of clinical detection of remodeling occurs in the acute and chronic phase of MI. Dilation of the infarcted area secondary to the expansion process may be found in the acute phase, and eccentric hypertrophy of infarcted area secondary to different stimuli may be detected in the chronic phase (Figure 1). Therefore, despite complex, post-infarction remodeling is clinically characterized by an increase in the ventricular size.^4,5^

Another diagnostic method, still not used in routine clinical practice, consists of the detection of cell markers, which is based on the fact that cardiac remodeling involves the reexpression of fetal genes. Several markers may indicate a remodeling process, including changes in the expression of myosin heavy chain isoforms, with an increase in alpha- and a decrease in beta-myosin heavy chain, increased expression of GLUT-1, alpha-actin, natriuretic peptide, galectin, caveolin, neuronal nitric oxide synthase, angiotensin-converting enzyme, a decrease in GLUT-4, SERCA2a, and a shift from glucose to fatty acid oxidation.^6-8^

## Clinical Implications

### Cardiac dysfunction

**Figure 2:**
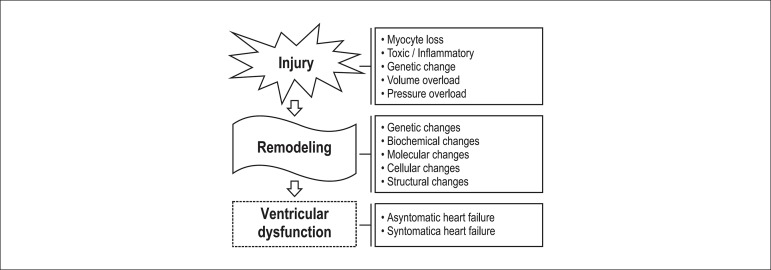
Sequence of events from cardiac injury to cardiac dysfunction.

Cardiac dysfunction is the main consequence of cardiac remodeling, which consists of a pathophysiological substrate for the onset and progression of ventricular dysfunction. This interaction starts with genetic changes in response to a cardiac injury, with reexpression of fetal genes. Consequently, cellular and molecular changes occur, resulting in progressive loss of ventricular function, asymptomatic at first, that evolves to signs and symptoms of heart failure^4-6,9^ (Figure 2).

An important aspect to be considered is that the occurrence of ventricular dysfunction has an impact on prognosis. Approximately 50% of patients with the diagnosis of cardiac dysfunction will die within five years. In addition, 40% of patients die within one year after hospitalization for cardiac failure.^10^ A significant part of deaths associated with cardiac remodeling/dysfunction is caused by sudden death,^11^ indicating that the fact that a patient is asymptomatic is not a guarantee of good prognosis. Despite increased survival with modern therapies, mortality rates are still at unacceptable levels.^12^

### Arrhythmias

It is well established that cardiac remodeling is associated with malignant ventricular arrhythmias, including sustained ventricular tachycardia and ventricular fibrillation. This is caused by different changes that will be discussed below.

The first mechanism involves ion channel changes, including inactivation of sodium channels, changes in calcium and potassium channels, and alterations in the sodium/calcium exchanger function.^13,14^

Another mechanism are changes in the gap junctional intercellular communication, responsible for the contact between adjacent cells, and hence, for the electrical coupling. Gap junction proteins are called connexins and the most prominent connexin expressed in the heart is the connexin 43. Whereas in the normal heart the connexin 43 is localized in the intercalated disc, a decrease in labeling intensity is observed in remodeling, in addition to a redistribution of the protein along the long sides of the cell. This process would lead to prolongation of the QT interval and arrhythmias.^13,14^

Finally, cardiac remodeling is associated with increased collagen content. Myocardial fibrillar collagen is divided into three compartments, the epimysium, perimysium and endomysium. The epimisyum ensheathes the entire muscle and constitutes the endocardium and the epicardium. From the epymisium, groups of muscle are wrapped and connected by the perimysium, and a thin layer of connective tissue derived from the perimysium, known as the endomysium, surrounds each of the muscle fibers and connects them to nearby capillaries. The increase in collagen content (fibrosis) involving these three components may cause blockage of electrical conduction and reentry arrhythmia. Therefore, fibrosis is associated with arrhythmias and sudden death, and strategies to reduce fibrosis, such as the use of angiotensin converting enzyme inhibitors, decrease the vulnerability to arrhythmias.^15^

### Myocardial infarction complications

During the first hours after coronary occlusion, disintegration of interfibrillar collagen may occur simultaneously with necrosis of myofibrils. The loss of sustaining tissue makes this area more susceptible to distension and deformation. Thinning of the infarcted region and dilation of the cavity occur as a consequence of slippage of necrotic muscle cells and rearrangement of the myocytes across the infarcted wall. This acute ventricular dilation, characterized by thinning and lengthening of the infarct is termed infarct expansion.^3^ Infarct expansion increases the likelihood of myocardial rupture and represents an anatomical substrate for aneurysms.^3^

## Pathophysiological Mechanisms of Cardiac Dysfunction

Although it is well known that ventricular remodeling leads to deterioration of ventricular function, the mechanisms underlying this phenomenon is not fully understood. Potential factors involved in this process are described in Table 1 and discussed below.

**Table 1:** Pathophysiology of ventricular dysfunction in cardiac remodeling

**Mechanism**	**Main changes**	**Consequence**
Cell death	↑ apoptosis, ↑necrosis ↓ autophagy	Progressive myocyte loss
Energy metabolism	β oxidation Triglyceride accumulation ↑ glycolysis Mitochondrial dysfunction Mitochondrial atrophy	Lipotoxicity ↓ energy ↑ oxidative stress
Oxidative stress	↑ NADPH oxidase ↑ catecholamine degradation ↑ xanthine oxidaseMitochondrial dysfunction ↓ antioxidant systems	Lipid peroxidation Protein oxidation DNA damage Cell dysfunction Fibroblast proliferation Metalloproteinase activation ↑ apoptosis ↑ signaling pathways to hypertrophy
Inflammation	innate response Adaptive response dysfunction	↑ inflammatory cytokines Macrophage, T cell and B cell dysfunction
Collagen	Fibroblast proliferation ↑ metalloproteinases	Degradation of normal collagen Fibrosis
Contractile proteins	β-myosin ↓ α-myosin ↑ troponin T type 2 ↓ troponin I phosphorylation	↓ contractility
Calcium transport	↓ L-type calcium channels ↓ ryanodine ↓ calsequestrin ↓ calmodulin ↓Phospholamban phosphorylation ↓ SERCA 2a	↓ Calcium in systole ↑ Calcium in diastole
Geometry	LV cavity ↓ wall thickness Elliptical shape → spherical shape	↑ parietal stress of the LV
Neurohormonal activation	↑ renin-angiotensin-aldosterone system ↑ Sympathetic	↑ cell death, ↑ oxidative stress, ↑ inflammation, ↑metalloproteinases and fibroblasts, hypertrophy, vasoconstriction

### Cell death

We can identify three main mechanisms involved in myocyte death: apoptosis or programmed cell death, necrosis and autophagy.

Previously, although the role of cell death on cardiac dysfunction progression was widely accepted, the exact involvement of apoptosis or necrosis in different models of cardiac injury was the subject of intense debate. However, recent evidence suggests that these mechanisms are closely related and may be different faces of the same process - necroptosis.^16^

Autophagy is an intracellular process characterized by the destruction of unnecessary or dysfunctional citoplasmatic components by lysosomes.^17^ Protein homeostasis, or proteostasis, depends on a delicate balance between protein synthesis, transport, post-translational modification and degradation. A disturbance on such balance may lead to accumulation of defective proteins and a process known as proteotoxicity. Therefore, autophagy exerts a crucial role in proteotoxicity prevention, with the participation of the ubiquitin system^17^ and chaperones, also known as heat shock protein-HSP.^18^ Recent evidence indicates that progression of ventricular dysfunction may be associated with changes in the process of autophagy, which can be either adaptive or deleterious.^16-18^

Therefore, despite different ways of cell death, the progressive loss of myocytes seems to play an essential role in remodeling, and a potential target for therapeutic interventions.

### Energy metabolism

Another factor potentially involved in alterations of the cardiac function after remodeling is energy deficit, resulting from the imbalance between oxygen supply and consumption. In normal conditions, free fatty acids are the major energy substrate for the heart, accounting for 60%-90% of energy supply. Fatty acid and glucose metabolites enter the citric acid cycle by β-oxidation and glycolysis, respectively, to generate FADH2 and NADH, which, in turn, participate in the electron transport chain. The generated energy is then stored and transported in the form of phosphocreatine.^19^

Altered energy metabolism has been reported in cardiac remodeling, with decreased free fatty acids oxidation and increased glucose oxidation. A decrease in β-oxidation may result in accumulation of triglycerides and lipotoxicity, and mitochondrial atrophy and altered mitochondrial function have been also described in cardiac remodeling. All these processes result in low energy availability for myocardial proteins with ATPase activity, and generation of reactive oxygen species, oxidative stress and its consequences.^20-22^

### Oxidative stress

Reactive oxygen species may be produced by several sources in the heart, including the mitochondrial electron transport chain, NADPH oxidase system, activity of the enzymes cyclooxygenase, cytochrome P450, glucose oxidase, xanthine oxidase, lipoxygenase, as well as by catecholamine degradation. In physiological conditions, there is a balance between reactive species production and antioxidant defense; the oxidative stress occurs when excess reactive oxygen species are generated that cannot be neutralized by antioxidant systems.^23^

Strong evidence supports an association between cardiac remodeling and oxidative stress resulting from increased reactive species production and decreased antioxidant defense. This would lead to several conditions, such as lipid peroxidation, protein oxidation, DNA damage, cellular dysfunction, proliferation of fibroblasts, activation of metalloproteinases, induction of apoptosis, changes in calcium-transport proteins, activation of hypertrophy signaling pathways, among others^24-26^. Therefore, the oxidative stress seems to play a significant pathophysiological role in cardiac remodeling.

### Inflammation

It is currently believed that both adaptive and innate immune responses are activated in response to cardiac injury. Whereas the innate system generates a more nonspecific inflammatory response, the adaptive system induces a more specific response, mediated by B and T cells.^27^

Experimental evidence has shown that inflammatory mediators induce the reexpression of fetal genes, cellular growth, activation of metalloproteinases, proliferation of fibroblasts, and progressive loss of myocytes by apoptosis. Similarly, antagonism of innate response (antagonists to toll-like receptors, TNF, IL-1 and IL-8) attenuated the cardiac remodeling after MI. Besides, modulation of the adaptive response (macrophages, regulatory T cells and B cells) may induce a more favorable remodeling, particularly in myocardial ischemia model.^27-29^

Therefore, although negative experiences with cytokine inhibitors have been reported in previous clinical trials, the inflammatory response remains as a potential target for therapeutic interventions.

### Collagen

There is a complex collagen network in the heart. The interstitium consists mainly (95%) of type I and type III collagen fibers. The main functions of this network are to regulate apoptosis, restore pathological deformations, maintain the alignment of structures, regulate the distensibility of the heart muscle and transmission of strength during fiber shortening, and express cytokines and growth factors.^30^

Collagen fibers are cross-linked by chemical bonds and are resistant to degradation of most proteases. Some enzymes, however, including metalloproteinases, have collagenolytic activity. The rupture of the collagen network could lead to several consequences for ventricular architecture and function. Therefore, in the acute MI model, increased metalloproteinase activity was associated with progressive ventricular dilation and cardiac dysfunction. The pharmacological inhibition of metalloproteinases has been shown to ameliorate cardiac remodeling.^31,32^

The abnormal accumulation of type III collagen and especially type I collagen (harder, longer and more stable) was detected in different models of cardiac injury, induced by several signaling pathways including TGF‑β, endothelin-1, angiotensin II, connective tissue growth factor, and platelet‑derived growth factor. In this context, fibrosis was associated with increased myocardial stiffness, diastolic dysfunction, weakened contraction, impaired coronary flow and malignant arrhythmias. In addition, fibrosis was a predictor of mortality in patients with cardiac dysfunction.^33,34^

Therefore, collagen plays a critical role in the maintenance of cardiac architecture and function. In the remodeling process, however, the balance between collagen synthesis and degradation may be affected with many adverse effects.

### Contractile proteins

Ventricular remodeling is characterized by alterations in the main contractile protein - myosin - composed of one pair of heavy chains (α and β) and two pairs of light chains. Depending on the myosin chain composition, three isomyosins (V1, V2 e V3) may be identified in the myocardium of different species. These isoenzymes possess the same pairs of light chains and differ by their heavy chain compositions (αα in V1, αβ in V2, and ββ in V3). The myosin ATPAase activity relies on active sites located on heavy chains, and α-fraction has the highest activity. The composition of isoenzymes, thus, determines the contractile capacity of myocytes. In addition to the predominance of the fetal form of myosin light chain, a decrease in V1 isoform accompanied by an increase in V3 isoform is commonly observed in remodeling. The relevance of this finding in rodent models has been questioned, since there is already a predominance of V3 isoform in humans. However, in cardiac remodeling and dysfunction, an additional decrease of V1 isoform has been reported in humans. In addition, increased troponin T type 2 and reduced phosphorylation of troponin I have been found after remodeling.^35,36^

### Calcium transport

Calcium transport through the sarcoplasmic reticulum is an active, complex process, involving many components. Membrane and intracellular systems (L-type calcium channels, ryanodine receptor, calsequestrin) regulate the supply of calcium to contractile proteins during contraction. Also, stimulation of calmodulin kinase and phosphorylation of phospholamban activates enzymes (SERCA-2a) that mediate calcium uptake by the sarcoplasmic reticulum, and enhances cardiac relaxation.^37^

Evidence suggests that alterations in the calcium transport system occur in ventricular remodeling and dysfunction, including a decrease in L-type calcium channels, and ryanodine receptors, and decreased calsequestrin and calmodulin kinase activity. Hence, cardiac remodeling leads to reduced calcium release during systole and increased release during diastole. Therefore, alterations in proteins involved in calcium transportation may contribute to cardiac dysfunction in remodeled hearts.^37,38^

### Changes in geometry

As previously described, cardiac remodeling is associated with changes in different mechanisms related to cardiac dysfunction. In some models, alterations in geometry, including changes in the wall thickness, cavity diameter, and normal configuration of the left ventricle (from elliptical to spherical), may lead to functional consequences. For example, in rat infarct models, the animals developed increased ventricular cavity associated with depressed global systolic function, and yet preserved myocyte contractile function.^39^ In aortic constriction model, nearly 50% of animals developed left ventricle dilation and pulmonary congestion, whereas the other group of animals had concentric hypertrophy, with no signs of pulmonary congestion. No differences in the contractile function were found between the groups. Thus, in some situations, change in geometry, *per se*, could affect the global ventricular function, by affecting cardiac load.^40^

Another relevant aspect of the role of geometry on cardiac function is the influence of ventricular rotation and torsion. The normal ventricular function requires coordination between electrical and mechanical activities. The left ventricular wall is first activated in the endocardial region of the septum and then on the ventricular free wall, from ventricular apex to the base, following the Purkinje fiber network. The mechanical response, however, is characterized by a physiological dyssynchrony between the subendocardial and subepicardial regions^41^.

"Rotation" is defined as a circumferential movement around the longitudinal axis. During isovolumetric contraction, the apex shows a brief clockwise rotation followed by a continued counterclockwise rotation during LV ejection. Parallel to this movement, a shortening of endocardial fibers and expansion of epicardial fibers occur, followed by simultaneous shortening of both types during ejection. In contrast, the base rotates counterclockwise and clockwise during isovolumetric contraction and ejection, respectively, to a lesser extent than the apex. The term torsion refers to the gradient between the base and the apex. Torsion, then, describes the degree of myocardial deformation, which is restored during diastole.^41^ The first consequence of systolic torsion is the increase in the intracavitary pressure with minimum shortening, which reduces the energy demand. In addition, torsion induces a more uniform distribution of LV fiber stress and fiber shortening across the wall. Also, the simultaneous presence of subendocardial and subepicardial vectors (i.e. shortening and lengthening vectors) during diastolic torsion, which initiates during isovolumetric relaxation, facilitates the recoil forces and restoration of ventricular architecture. Therefore, the loss of torsion affects systolic and diastolic function of the LV.^42^ In cardiac remodeling, changes in cardiac architecture may lead to alterations in torsion and result in cardiac dysfunction. In some situations, surgical intervention for ventricular restoration could be beneficial.^43^

### Neurohormonal activation

Two of the main systems involved in cardiac remodeling are the sympathetic system and the renin-angiotensin-aldosterone system. Activation of both systems activates intracellular signaling pathways that stimulate the synthesis of protein in myocytes and fibroblasts, causing cellular hypertrophy and fibrosis. Other effects reported include activation of growth factors and metalloproteinases, hemodynamic overload by vasoconstriction and water retention, increase in oxidative stress and direct cytotoxic effect, leading to cellular death by necrosis or apoptosis.^44-46^ Blockage of these systems has an important role in prevention or attenuation of cardiac remodeling secondary to stimuli.

## Pharmacological Treatment

**Figure 3:**
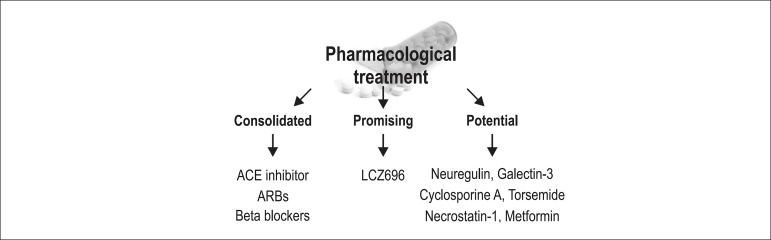
Pharmacological treatment of cardiac remodeling. ACE: Angiotensin-converting-enzyme; ARBs: Angiotensin receptor blockers.

Pharmacological treatment of cardiac remodeling can be divided by three strategy stages: consolidated, promising and potential strategies (Figure 3).

In the consolidated strategy group, angiotensin-converting enzyme inhibitors, beta blockers, and aldosterone antagonists have been consistently shown to decrease remodeling in animal models.^44-47^ These findings have been validated in clinical trials, and these drugs are currently indicated for patients with ejection fraction of < 40%.^47^

LCZ696 stands out among the promising strategies. LCZ696 combines a valsartan molecule (angiotensin II receptor antagonist) and sacubitril (inhibitor of neprilysin, which metabolizes natriuretic peptides, urodilatin, bradykinin and adrenomedullin). Experimental studies showed attenuation of ventricular cavity dilation and myocardial fibrosis after MI, for example.^48^

Results from experimental studies served as the basis for the development of a big clinical trial, the PARADIGM-HF trial^49^. More than 8,000 patients with symptomatic chronic heart failure (NYHA class II-IV) and drop in ejection fraction were randomized to receive either LCZ696 or enalapril. After a follow-up of 27 months, the LCZ696 showed lower all-cause mortality rate, lower cardiovascular mortality, and fewer hospitalizations for cardiac failure.^49^ LCZ696 may change the current treatment of patients with symptomatic systolic heart failure.

With respect to potential therapies, the main targets are pathophysiological mechanisms previously described, especially in experimental studies. Cell death has been one of the main targets investigated. Previous studies have shown that cyclosporine and neuregulin-1 attenuate apoptosis; also, necrostatin-1 attenuates apoptosis via inhibition of caspase-8 and reduces necrosis via blockage of calpain activity. Modulation of chaperones and the ubiquitin‑proteasome system (hence modulating protein degradation) would also lead to greater survival.^50^ Fibrosis has also been an attractive target for therapeutic interventions. Inhibition of thrombospondin-1 and galectin-3 is associated with a decrease of collagen content.^51^ The same effect was reported after administration of torsemide and metformin.^50^ In addition, administration of CXL-1020, a nitroxyl donor, enhanced the sensitivity of contractile proteins to calcium, with consequent functional improvement and attenuation of hypertrophy.^50^ Also, modulation of inflammatory process, including macrophages, T lymphocytes and cytokines has been investigated in different models, with promising results.^52^

Continuous investigation of new compounds for the attenuation of cardiac remodeling/dysfunction has been made, and a number of potential strategies are currently available.

## Conclusion

Cardiac remodeling is associated with the development and progression of ventricular dysfunction, arrhythmias and poor prognosis. After MI, may predispose to ventricular rupture and aneurysm formation. Despite therapeutic advances, mortality rates related to cardiac remodeling/dysfunction remain high. Therefore, the understanding of the pathophysiological mechanisms involved in remodeling process is crucial, including to develop new therapeutic strategies.
